# Treatment of Itch in Belgium

**Published:** 1845-04

**Authors:** 


*Treatment of the Itch in Belgium.*—The following circular has been addressed to military surgeons by the inspector-general of the Belgian army:

Each patient is to be supplied with an ounce or an ounce and a half of liquid sulphuret of lime in a small pot; this quantity he is to rub carefully and slowly with his hands on every part that is covered with papulæ. If there be any papulæ on the back, another patient is to rub the liquid upon that part. The operation is to be repeated three times in the twenty-four hours, so that each patient consumes three or four ounces of the sulphuret daily. A bath is to be taken every alternate day; the frictions are to be suspended on that day. Fifteen frictions (or ten days' use) are usually sufficient for the cure of the disease, if the medical officer in charge sees that the remedy is properly used.

The sulphuret is prepared thus: take of sublimed sulphur 16 pounds, and of quick lime 32 pounds; boil in 80 pounds of water for three-quarters of an hour. Let the mixture rest for some time until it settles, and then let the clear fluid be decanted off. Boil the residue again in about the same quantity of water, treat it in a similar manner, and add this decoction to the first. Usually 140 pounds of the sulphuret, at 12° by the areometer, are thus obtained. If the liquid be more dense it should be lowered to this standard by the addition of rain water.—*Braithwaite's Retrospect of Practical Med. and Surg.*

